# Supplementary material for: Mechanisms and contextual factors related to key elements of a successful integrated community-based approach aimed at reducing socioeconomic health inequalities in the Netherlands: A realist evaluation perspective
Source: PLoS One. 2023 May 17;18(5):e0284903. doi: 10.1371/journal.pone.0284903 (PMC10191363; doi:10.1371/journal.pone.0284903)
Supplement: S1 File — CMO configurations of the Zwolle Healthy City approach. (DOCX) [file pone.0284903.s001.docx]

**Repository:** Context-mechanism-outcome configurations of the Zwolle Healthy City approach.

**Table 1. Definitions, operational description and elaboration of ‘context’, ‘mechanism’ and ‘outcome’.^[[1]](#footnote-2)^**

|  | **Definition** | **Operational description** | **Elaboration** |
| --- | --- | --- | --- |
| **Context** | For who and in what circumstances does the mechanism work. A relationship between mechanisms and their effects is not fixed but contingent, depending on the context. | Something happening outside control of the Zwolle Healthy City approach, but of influence on the outcome. | A contextual factor can be   - Situational: in a specific moment in time   Or   - Continuous: ongoing   And   - Supportive to the mechanism   Or   - Restraining to the mechanism   E.g. policy, (financial) resources, organizational factors |
| **Mechanism** | Responsible for the relationship between context and outcome. The cause of change. | The response that actors have to activities or actions in the Zwolle Healthy City approach that, in the context, contribute to the outcome. | Activities and actions taken by professionals on the   - strategic level - tactic level - operational level   of involved organizations. |
| **Outcome** | Result of the combination of a mechanism with one or more supportive or restraining contextual factors. | Key elements of Zwolle Healthy City. | Nine key elements that were previously described by Wilderink et al. (2020). |

| **Outcome 1: Collaboration between a variety of local organizations** |
| --- |
| **CMO configuration 1.1**  **Mechanism: Formulating goals together increases for involved processionals the feeling of being a part of the approach and wanting to work together** Formulating goals for the approach together at the tactical level (managers from policy, practice and research) contributes positively to collaboration between a variety of local organizations. This also involves regularly checking whether everyone is still aware of the same goal.  *Context: Good relations (continuous, supportive)*  A contextual factor that reinforces this mechanism is good relationships between the persons representing the network organizations. Good personal relationship makes collaborating easier. |
| **CMO configuration 1.2**  **Mechanism: Identifying and clarifying roles and responsibilities of each partner organization makes the involved professionals aware of what their task is**  Jointly identifying and clarifying roles and responsibilities of each partner organization at the tactical level contributes positively to collaboration, because this provides clarity of what part of the implementation this local organizations is responsible for and where they contribute.  *Context: Roles and responsibilities align with goals and structures of the individual organizations (continuous, supportive)*  A supporting contextual factor is that the roles and responsibilities that are defined align with the goals and structures of the individual organizations. For example, in a sport activity for youth, the municipal sport service organization is responsible for the organization of the sport tournament, and the local welfare organization is responsible for the recruitment of participants, because the latter is known for easily making contact with citizens. |
| **CMO configuration 1.3**  **Mechanism: Meeting regularly to exchange knowledge and coordinate implementation makes that involved professionals see the advantage of collaboration**  The regularly (monthly) meetings of professionals working at the tactical level of involved organizations makes a positive contribution to collaboration, because in those meetings the implementation of the approach (at the operational level) is coordinated, and knowledge is exchanged.  *Context: Local existing structures (continuous, supportive)*  This mechanism is possible in the context of available local structures in the Netherlands in which the local organizations already function and depend on each other. Multiple organizations who work in the same neighborhoods are held accountable for the same ambitions by the local government, for example the wellbeing of citizens. Every organization benefits from collaborating and combining different expertise’s. |
| **CMO configuration 1.4.**  **Mechanism: Coordinating the approach and the network by a program manager leads to more clarity among involved professionals about their specific tasks**  Coordinating the approach and the network of involved organizations at the tactical level by a program manager makes a positive contribution to collaboration. Coordination is about monitoring progress and facilitating or stimulating the (joint) implementation by the organizations involved.  *Context: Financial resources (continuous, supportive)*  A reinforcing contextual factor is that there are financial resources for appointing this program manager, for example provided by the local government. |
| **CMO configuration 1.5**  **Mechanism: Designing local policy in an integrated way creates a sense of urgency to collaborate with other domains at the tactic and operational level**  Designing local policy in an integrated way is a mechanism related to collaboration between a variety of local organizations. When local health policy is designed at the strategic level in collaboration with different domains (e.g. policies aimed at reducing poverty, health and wellbeing of youth or urban planning), this contributes positively to collaboration between the involved network organizations. It stimulates to collaborate with organizations from other policy domains.  *Context: (new) laws that stimulate integrated policy (situational, supportive)*  The new Dutch Child and Youth Act (Jeugdwet 2015) and Environment and Planning Act (Omgevingswet 2021) are new laws that, in theory, may stimulate integrated policy and can be seen as contextual factors that reinforce this mechanism. |
| **CMO configuration 1.6**  **Mechanism: Facilitating and stimulating collaboration at the operational level makes professionals working on this level feel more motivated to collaborate**  Decisions at the tactical level to make time, money, resources and support available for implementation of the approach contribute to good cooperation at the operational level.  *Context: Involved organizations assign professionals at the operational level to a neighborhood/area*  Linking professionals to an area or neighborhood, instead of linking the professional to a role or task, supports collaboration at the operational level between a variety of organizations. Through this, professionals from different involved organizations always work in the same neighborhood, know each other, and can carry out activities and projects together. For example, a welfare worker is not responsible for sociocultural work in the entire municipality, and another colleague for sports, but the welfare worker is responsible for sociocultural work, sports, creative activities, etc. in a specific neighborhood.  *Context: Overlap in the objectives of involved organizations (continuous, supportive)*  A contextual factor that works supportive in this, is that there is overlap in the objectives of involved organizations. Because of this, it makes sense to carry out activities and projects in the area or neighborhood together with professionals of other organizations. |
| **Outcome 2: Support on three levels: Strategic, tactical and operational** |
| **CMO configuration 2.1**  **Mechanism: Having regularly meetings with involved aldermen makes that involved professionals feel like they have a say in local policy making**  Regular meetings of the network of involved organizations with involved aldermen contributes positively to support at the tactical level. Through those meetings, once or twice a year, involved organizations felt like they could influence policy measures made by the aldermen.  *Context: Alderman embraces approach (situational, supportive)*  This works well if the responsible alderman embraces the approach and is willing to take the visions of involved organizations into account. |
| **CMO configuration 2.2**  **Mechanism: Assigning professionals that function as a bridge between the tactical level (managers) and operational level leads to clarity among operational professionals about their tasks and roles**  A group of professionals that function as a bridge between the tactical and operational level contribute positively to support at the operational level. Those professionals work for the municipal health services and have the task to convert the policy into neighborhood-specific practices, and the other way around. As a consequence, the vision of Zwolle Healthy City was successfully translated to a realistic and endorsed activity program, which contributed to support at the operational level.  *Context: Financial resources available to assign professionals as ‘bridge’ (continuous, supportive)* |
| **CMO configuration 2.3**  **Mechanism: Aligning approach with daily work of professionals and at the same time see the added value makes that professionals do not feel obligates to do extra work**  This contributes to support at the operational level. By aligning the approach with their daily work, professionals of involved organizations did not feel obligated to do extra activities for Zwolle Healthy City, which increased their support.  *Context: Work agreements between local government and implementing organizations are in line with approach (situational, supportive)*  What works supportive to this mechanism is when work agreements between local government (the financing organization) and implementing organizations are in line with aims and goals of the approach. In this way, organizations are obligated to work on the theme ‘healthy lifestyle’ irrespective of the motivation of the person responsible for the implementation. |
| **CMO configuration 2.4**  **Mechanism: Assigning internal coordinator at the operational level who functions as an ambassador within their own organization makes that professionals feel more urge and support to work on the goals of Zwolle Healthy City**  Those internal coordinators are responsible for generating attention for the Zwolle Healthy City approach among their colleagues, the coordination of the implementing and embedding the approach in their organization. This contributes to support at the operational level.  *Context: Financial resources for internal coordination available (continuous, supportive)* |
| **CMO configuration 2.5**  **Mechanism: Making clear that health promotion transcends domains and that the entire local board of Mayor and Aldermen must support the approach leads to the awareness among the board that they also have a role in the approach**  Socio-economic health inequalities are a complex problem and the solution therefore transcends domains. Support at the strategic level can be promoted when this is clear for the entire local board of Mayor and Aldermen.  *Context: Enthusiastic alderman (situational, supportive)*  An enthusiastic alderman can inspire other aldermen, which contributes positively to broad support for the approach in the entire local board of Mayor and Aldermen. |
| **CMO configuration 2.6**  **Mechanism: Communicating extensively (internal and external) and celebrating successes together makes that professionals see what the approach brings and with that increase support**  Making clear and visible what happens within the approach, in terms of processes and activities, contributes to support among colleagues at strategic, tactical and operational level.  *Context: Cost-benefit analysis of prevention is difficult (continuous, restraining)*  This makes showing the success of the approach difficult. This is especially important at a strategic level.  *Context: Monitoring and evaluation (situational, supportive)*  Monitoring and evaluation makes it possible to show the impact of the approach. |
| **CMO configuration 2.7**  **Mechanism: Framing the approach in a positive way makes that people want to be a part of the approach**  No one is against promoting health and people are in general willing to support improving health. That’s why it’s important to frame the approach in a positive way, for example by calling the approach ‘Zwolle HEALTHY City’. It’s not helpful to frame the approach in negative health problems.  *Context: Local policy aims to reduce socioeconomic health inequalities* |
| **Outcome 3: Coordination and communication** |
| **CMO configuration 3.1**  **Mechanism: Assigning a program manager that coordinates the network of involved organizations leads to more clarity among involved professionals about their specific tasks**  Assigning a program manager who chairs the network contributes positively to the outcome of coordination and communication. A program manager is responsible for communicating about the meetings at the tactical level and coordinating the implementation and assignments of tasks for each organization. For example, the monitoring and evaluation of the approach is coordinated by the program manager and the involved professor of the University of Applied Sciences.  *Context: Financial resources for program manager available (continuous, supportive)*  *Context: Program manager works for the local government and is physically present at the town hall (situational, supportive)*  In the period that the Zwolle Healthy City approach was implemented, the program manager was for the first couple of years occupied at the local government. This was seen as a supportive contextual factor for those first years, because at the local government, this professional is physically close to the policy makers (i.e. in the same building). This makes it easier to bring different policy domains together.  *Context: Program manager works for the municipal health service organization (situational, supportive)*  For the years after, since 2014, the program manager was occupied at the regional public health service organization (‘GGD’ in Dutch). An occupation at the regional public health service organization is supportive as well, because there’s expertise on health promotion there. |
| **CMO configuration 3.2**  **Mechanism: Assigning professionals that function as a bridge between the tactical level (managers) and operational level leads to clarity among operational professionals about their tasks and toles**  A group of professionals that function as a bridge between the tactical and operational level contribute positively to the key element of coordination and communication. Those professionals were working for the municipal health services and had the task to convert the policy into neighborhood-specific practices, and the other way around. As a consequence, the vision of Zwolle Healthy City was successfully translated to a realistic and endorsed activity program, which contributed to support at the operational level.  *Context: Financial resources for professionals that function as bridge available (continuous, supportive)* |
| **CMO configuration 3.3**  **Mechanism: Assigning internal coordinators at the operational level of involved organizations makes that professionals feel more urge and support to work on the goals of Zwolle Healthy City**  Those internal coordinators are responsible for generating attention for the Zwolle Healthy City approach among their colleagues, the coordination of the implementing and embedding the approach in their organization. This contributes to support at the operational level.  *Context: Financial resources for internal coordinators available (continuous, supportive)* |
| **Outcome 4: Embeddedness of the approach in organization’s policy and processes** |
| **CMO configuration 4.1**  **Mechanism: Integrating the theme of the Zwolle Healthy City approach, ‘healthy lifestyle’, in regular activities of involved organizations makes that professionals do not feel they have to do something in addition to their regular work**  Integrating the theme of the Zwolle Healthy City approach facilitates embeddedness of the approach in the organization’s processes. If the involved organizations integrate the healthy lifestyle theme into the activities they already carry out, it is easier to embed the approach. It works positive to align the approach with the daily work of professionals at the operational level. In this way the professionals do not experience the feeling of having to do something in addition to their regular work, and it is also easier to keep doing this in the long term. For example, in all activities of the welfare organization, attention is now paid to healthy drinks and healthy snacks.  *Context: Involved organizations don’t see health as part of their core business (continuous, restraining)*  If it is not clear to professionals how the healthy lifestyle theme can be integrated into their daily work, this works restraining for embedding. The welfare organization believes that they are responsible for the welfare; the sports service organization believes that they are responsible for sports. If the goals of Zwolle Healthy City are far removed from these basic core tasks, it is more difficult to embed the approach. |
| **CMO configuration 4.2**  **Mechanism: Including the theme ‘healthy lifestyle’ in organizations’ written policy and in established agreements between the local government (financing organization) and organizations responsible for implementing the approach makes that professionals are obligated to work on the theme ‘healthy lifestyle’**  Including the theme ‘healthy lifestyle’ in the policy of organizations and in formal agreements between the local government (financing organization) and executive organizations contributes positively to embeddedness of the approach. In this way, organizations are obligated to work on the theme ‘healthy lifestyle’ irrespective of the motivation of the person responsible for the implementation.  *Context: Local policy aims to promote a healthy lifestyle* |
| **Outcome 5: Collaboration with private organizations** |
| **CMO configuration 5.1**  **Mechanism: Exploring opportunities for their role in the approach together with potential private organizations increases their motivation to contribute**  This contributes positively to collaboration with private organizations, because trough this jointly exploring, private organizations come up with ideas themselves that are in line with both their mission and goals, and the mission and goals of the approach.  *Context: Differences in culture, language and interest between public and private organizations (continuous, restraining)* |
| **CMO configuration 5.2**  **Mechanism: Asking private organizations to collaborate for a specific goal or task makes it easier for these organization to contribute**  It can also work positively to collaboration to ask private organizations for a specific goal or task in the approach.  *Context: Local policy aims to reduce socioeconomic health inequalities* |
| **CMO configuration 5.3**  **Mechanism: Collaborating with *local* private organizations ensures that they can take a specific practical role in the implementation of the approach**  Working with *local* private organizations can contribute positively to the outcome of collaborating with private organizations. Local organizations could, in contrast to national organizations, play a specific role in the implementation of the approach. The local supermarket for example is able to provide free fruit for the participating children of an organized sports tournament.  *Context: Local organization feel involved in their neighborhood (continuous, supportive)*  Collaborating with local private organizations works well in the context where local organizations feel involved in their neighborhood and recognize the importance of promoting a healthy lifestyle for their customers and employees. |
| **Outcome 6: Collaboration with citizens** |
| **CMO configuration 6.1**  **Mechanism: Giving citizens responsibilities in the organization of an activity makes citizens more motivated to be actively involved in the implementation of the approach**  Giving citizens responsibilities increases the willingness to participate. By making citizens responsible for a task, they feel that they are taken seriously and they are more willing to participate. For example, youths can be responsible for organizing a sport event or parents can be responsible for food/drink at an activity.  *Context: Professionals have time for coordination, financial resources available (continuous, supportive)*  This works well in a context where professionals have time to coordinate the participation of citizens. |
| **CMO configuration 6.2**  **Mechanism: Involving citizens in a project or activity right from the start prevents that citizens feel like things are already decided upon which can make them feel unimportant**  If professionals at the operational level are involving citizens from the start, activities are demand-oriented and align with the preferences and living conditions of the citizens.  *Context: Welfare organizations are well organized in the municipality, supporting existing infrastructure (continuous, supportive)*  Welfare work is about being present: community workers are present on the streets and know the citizens and know what their preferences are. As a result, they offer activities and services that meet the needs of the target group. These needs can be different for every neighborhood. |
| **CMO configuration 6.3**  **Mechanism: Investing in trust by taking time to listen to citizens’ opinions and include these in further development and implementation improved the trust relationship with citizens**  Having a conversation with citizens and taking the time to listen to them creates trust and contributes to good collaboration with citizens in the long term.  *Context: (senior) citizens often consider trust important and change difficult* |
| **CMO configuration 6.4**  **Mechanism: Using existing social infrastructure in the neighborhood as existing groups and ambassadors makes it easier for professionals to reach low SEP groups**  By making use of groups that already exist, it is easier to reach citizens in low SEP neighborhoods who do not have improving their health as a first priority. By using existing groups or ambassadors you can reach those groups.  *Context: People with low SEP are relatively hard to reach and involve (continuous, restraining)*  People with a relatively low SEP are in general not easy to reach groups, what makes collaboration more difficult. |
| **CMO configuration 6.5**  **Mechanism: Making the option to participate easy and accessible for citizens makes that citizens are more willing to participate**  Citizens are reached by making the range of activities as accessible and easy as possible, tailored to the wishes and needs of the target group.  *Context: Low SEP target group has more difficulties in understanding (e.g. because of migration background or low literacy) (continuous, restraining)* |
| **Outcome 7: Profiling the approach like a brand** |
| **CMO configuration 7.1**  **Mechanism: Linking successes of involved organizations to the Zwolle Healthy City approach makes that professionals see the added value of contributing to the approach**  By linking the successes to the approach, people and organizations become familiar with the approach and see the added value of contributing. This can lead to new collaboration opportunities and support for the approach.  *Context: Involved organizations see the approach as embedded in their own organization (situational, restraining)*  If involved organizations see the approach as completely embedded in their own organization (embedded in their activities and policies), they prefer to communicate as if the successes come from them, and not from the Zwolle Healthy City approach. |
| **CMO configuration 7.2**  **Mechanism: A figurehead or ambassador spreads the positive ambition of the Zwolle Healthy City approach and with that mobilizes others**  A figurehead or ambassador can contribute positively to profiling the approach, because it has more impact if a well known person spreads the ambition. People tend to listen to well known people.  *Context: Locally famous persons are used as figureheads or ambassadors (situational, supportive)*  A (locally) famous person has a large reach inside and outside the municipality. |
| **Outcome 8: Move along with, and take advantage of, (local and national) opportunities** |
| **CMO configuration 8.1**  **Mechanism: Making use of national funding opportunities increases the possibilities for professionals for local initiatives**  This provides opportunities for continuing local activities and interventions.  *Context: National attention for a healthy lifestyle (for example the ‘Healthy school approach’ or ‘Everything is health’ movement) (situational, supportive)* |
| **CMO configuration 8.2**  **Mechanism: Building on existing structures makes collaboration for professionals of different organizations easier because they are already familiar with each other**  Making use of existing structures facilitates starting working together on a new approach.  *Context: ‘Healthy Together’ was an ongoing existing structure in Zwolle (situational, supportive)*  ‘Healthy Together’ was an collaboration between (a part of) involved organizations, which made the collaboration at the start of Zwolle Healthy City in 2010 easier. |
| **Outcome 9: Continuous monitoring and evaluation goals and process, and learning from the results** |
| **CMO configuration 9.1**  **Mechanism: Making results and conclusions of evaluation clear and communicate about this towards other involved organizations makes professionals more willing to contrinute to the approach**  If researchers show the results and conclusions of monitoring and evaluation, involved organizations that implement the approach can see the added value of it. They can see what monitoring and evaluation can yield. This makes them more willing to participate in research.  *Context: Gap between research institutions and implementing organizations (continuous, restraining)*  The goal of monitoring and evaluation should be clear for everyone, but this is not always the case for implementing organizations  *Context: A research institution is situated in the municipality (situational, supportive)*  In Zwolle, a University of Applied Sciences is involved as a partner within the approach and is responsible for monitoring and evaluation. This makes monitoring and evaluation possible. |
| **CMO configuration 9.2**  **Mechanism: Drawing conclusions from monitoring and evaluation and adapt the approach based on those conclusions increases the support for monitoring and evaluation because professionals see that it actually helps in further developing the approach**  Drawing conclusions from monitoring and evaluation is important for a learning approach as Zwolle Healthy City, because this makes learning from the results and further development of the approach possible. Moreover, seeing that the approach is adapted based on the conclusions makes professionals more willing to contribute to monitoring and evaluation in the future.  *Context: Involved organizations do not hold each other accountable for the results of monitoring and evaluation (continuous, restraining)*  There is no reckoning between involved organizations, which works restraining for drawing conclusions on (negative) evaluations. |
| **CMO configuration 9.3**  **Mechanism: Using exiting monitoring data to evaluate the approach can make monitoring and evaluation easier for researchers**  Using existing monitoring data contributes positively to this outcome of monitoring and evaluation, because this can make monitoring easier for researchers as they do not have to collect data themselves.  *Context: The local government and the regional public health service organization have data available at municipal and neighborhood level (situational, supportive)* |

1. Based on Byng et al., 2005; Herens et al., 2017; Pawson, 2002; Pawson & Tilley, 1997 [↑](#footnote-ref-2)
