# Supplementary material for: Mechanisms and contextual factors related to key elements of a successful integrated community-based approach aimed at reducing socioeconomic health inequalities in the Netherlands: A realist evaluation perspective
Source: PLoS One. 2023 May 17;18(5):e0284903. doi: 10.1371/journal.pone.0284903 (PMC10191363; doi:10.1371/journal.pone.0284903)
Supplement: S3 File — Mechanisms and contextual factors related to key elements of Zwolle Healthy City. (PDF) [file pone.0284903.s003.pdf]

Mechanisms and contextual factors related to key elements of Zwolle Healthy City

A local integrated approach aimed at reducing socioeconomic health inequalities in the Netherlands

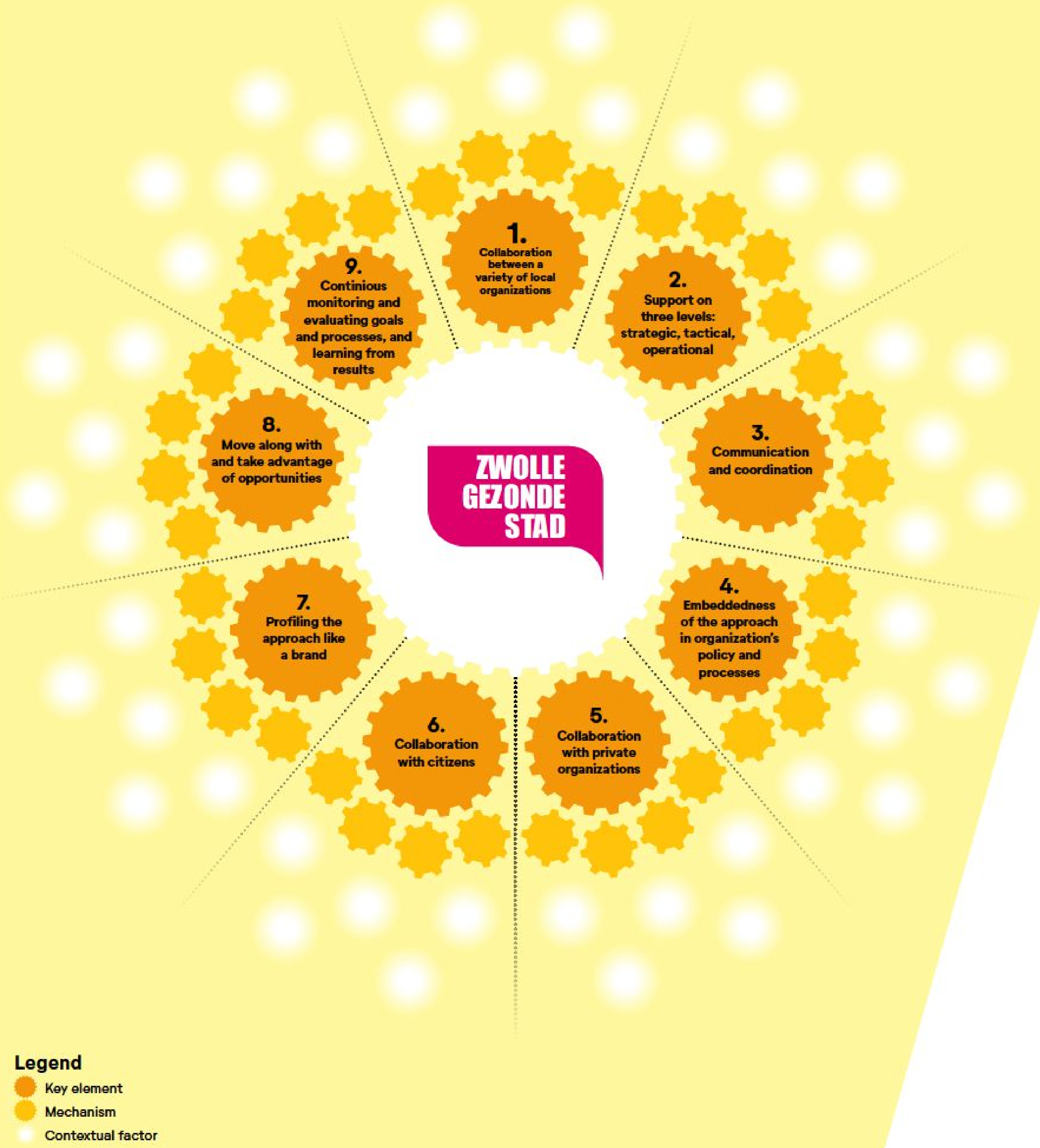

Wilderink et al. (2022)

1. Collaboration between a variety of local organizations

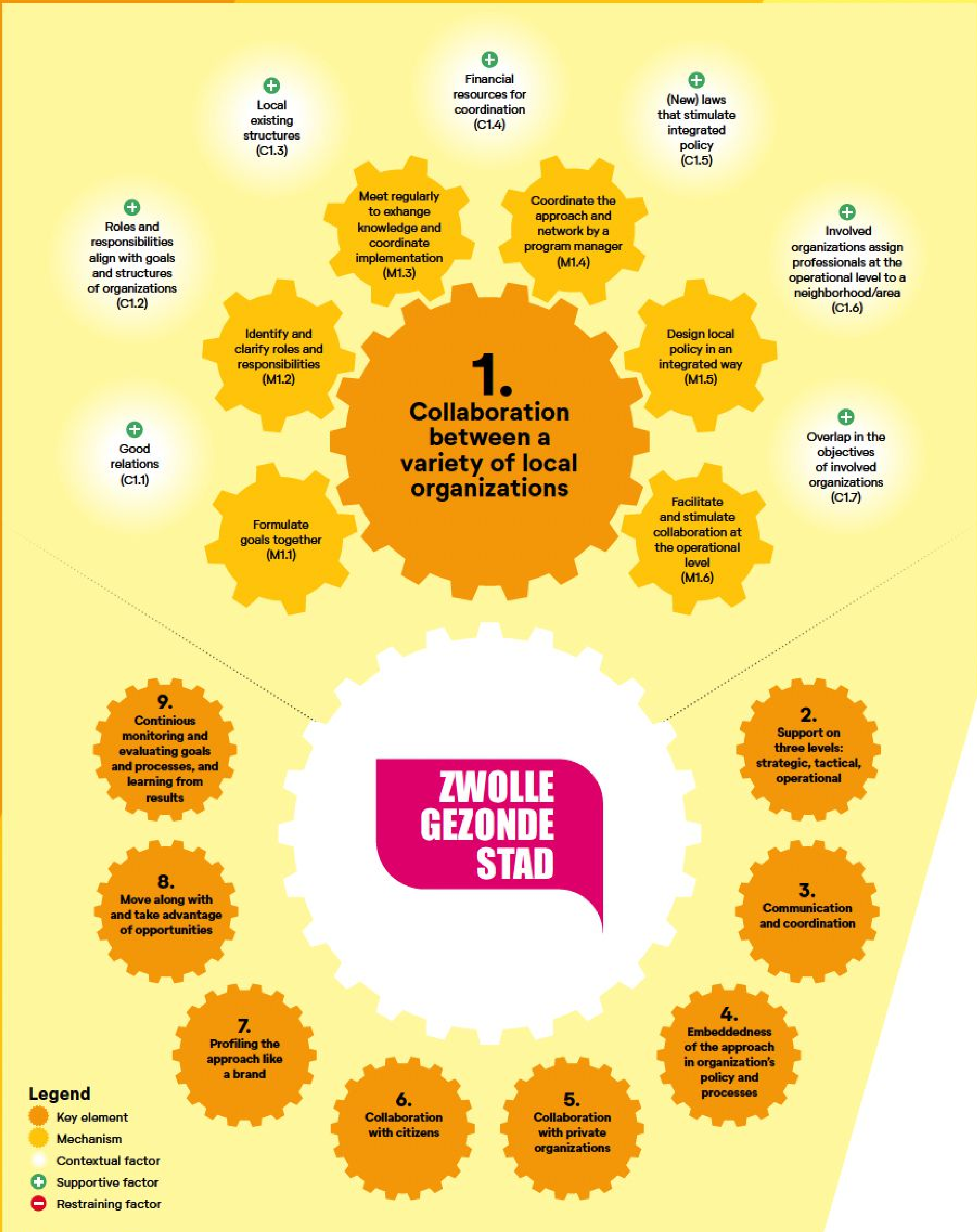

Wilderink et al. (2022)

2. Support on three levels: strategic, tactical, operational

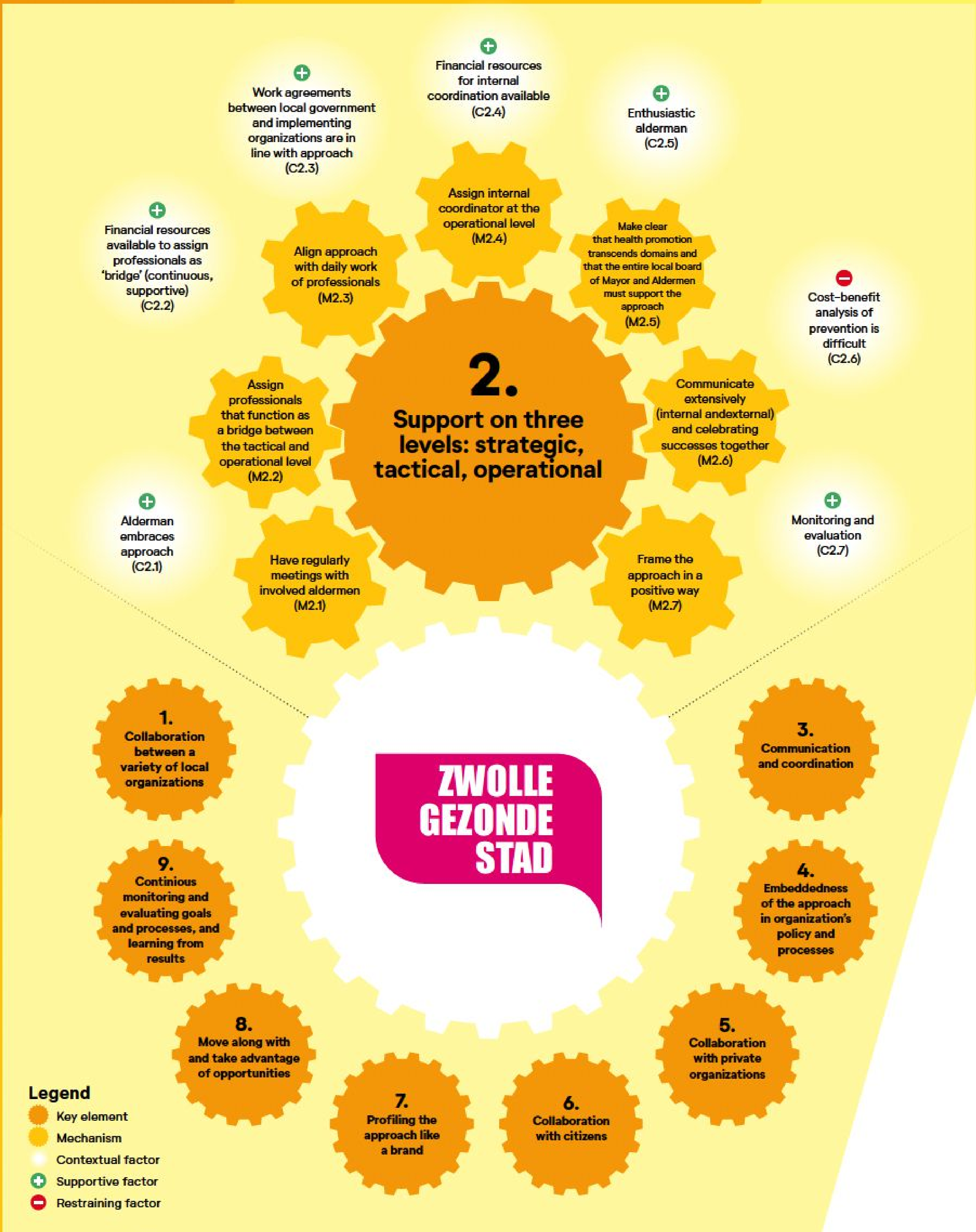

Wilderink et al. (2022)

3. Communication and coordination

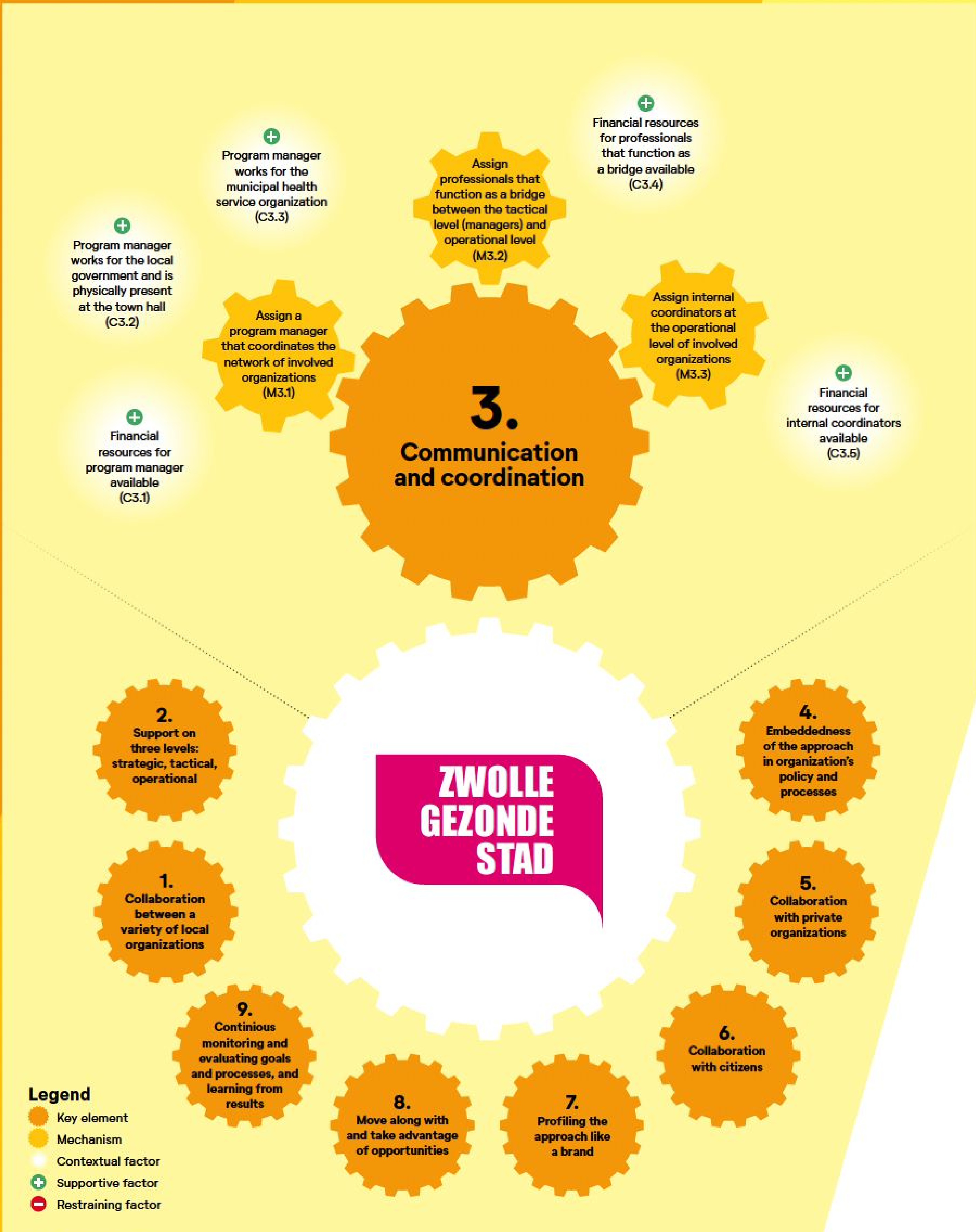

Wilderink et al. (2022)

4. Embeddedness of the approach in organization's policy and processes

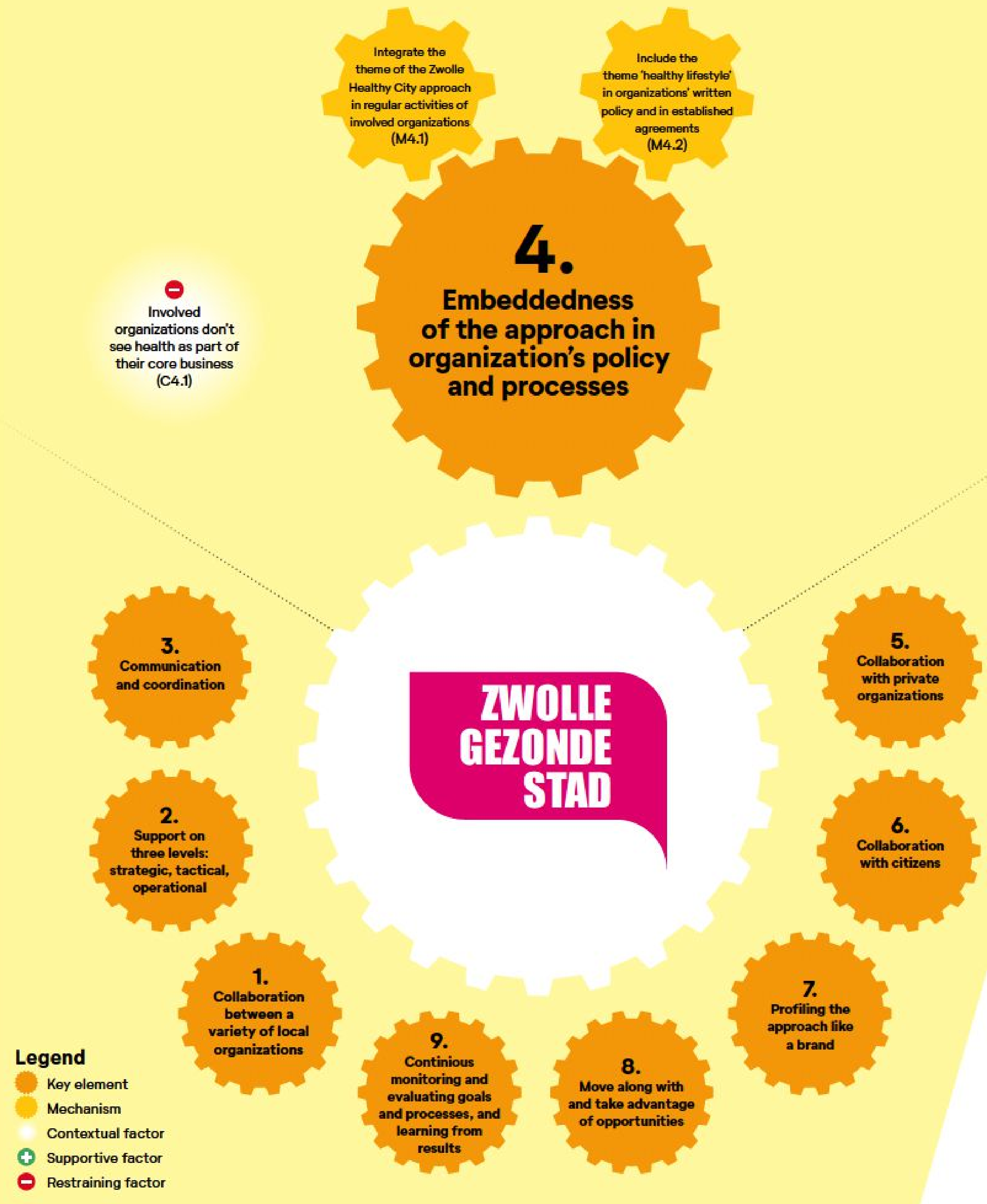

Wilderink et al. (2022)

5. Collaboration with private organizations

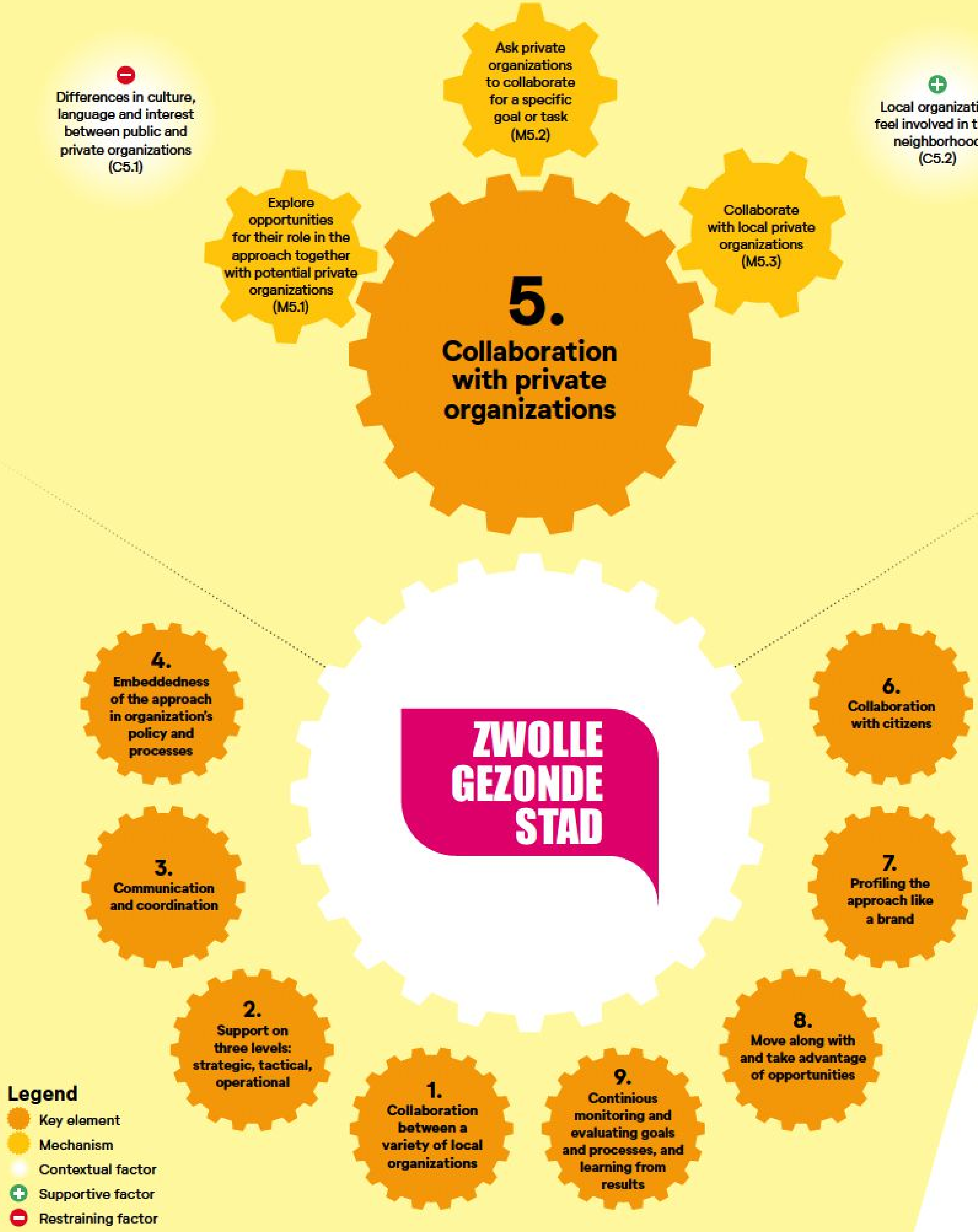

Wilderink et al. (2022)

6. Collaboration with citizens

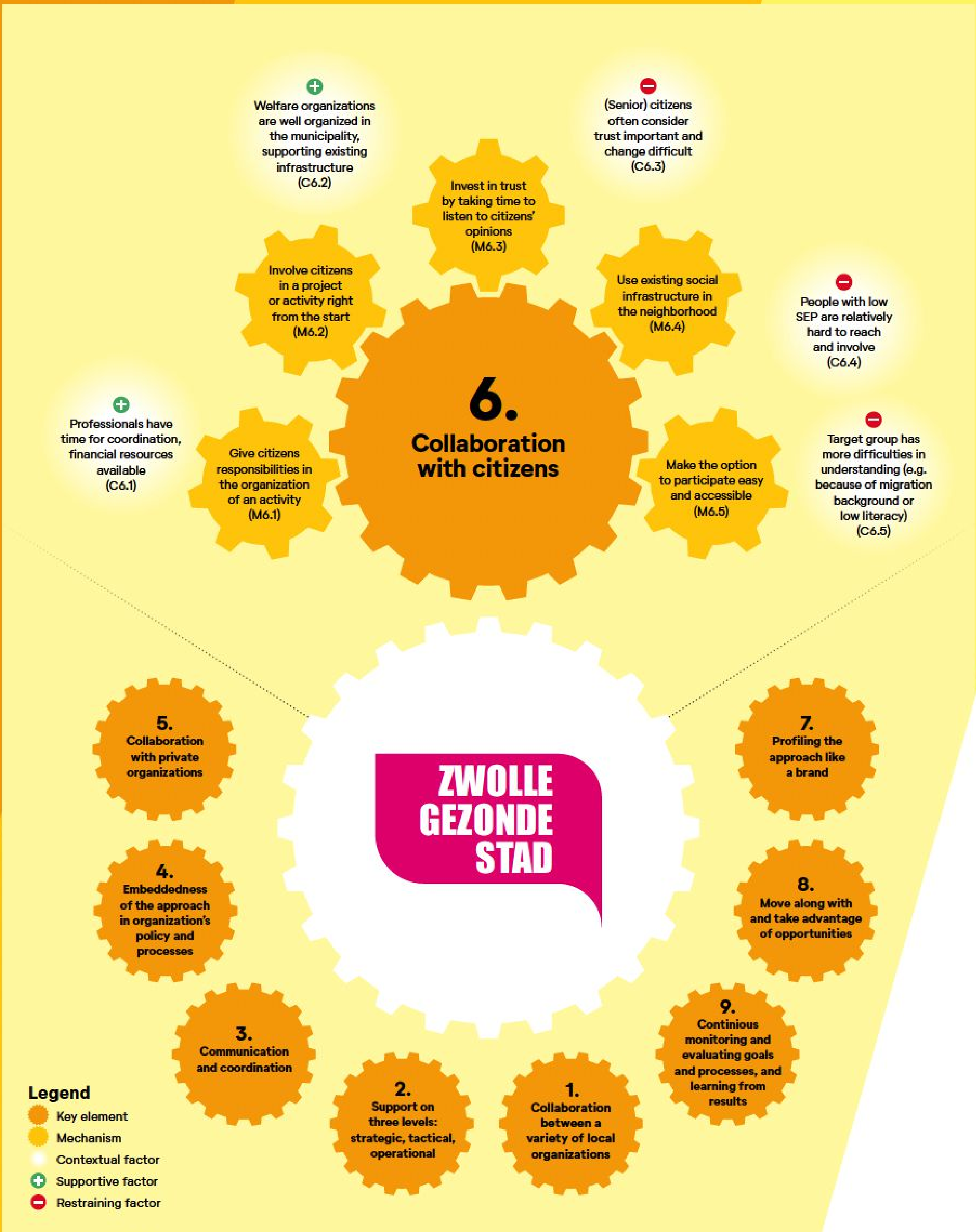

Wilderink et al. (2022)

7. Profiling the approach like a brand

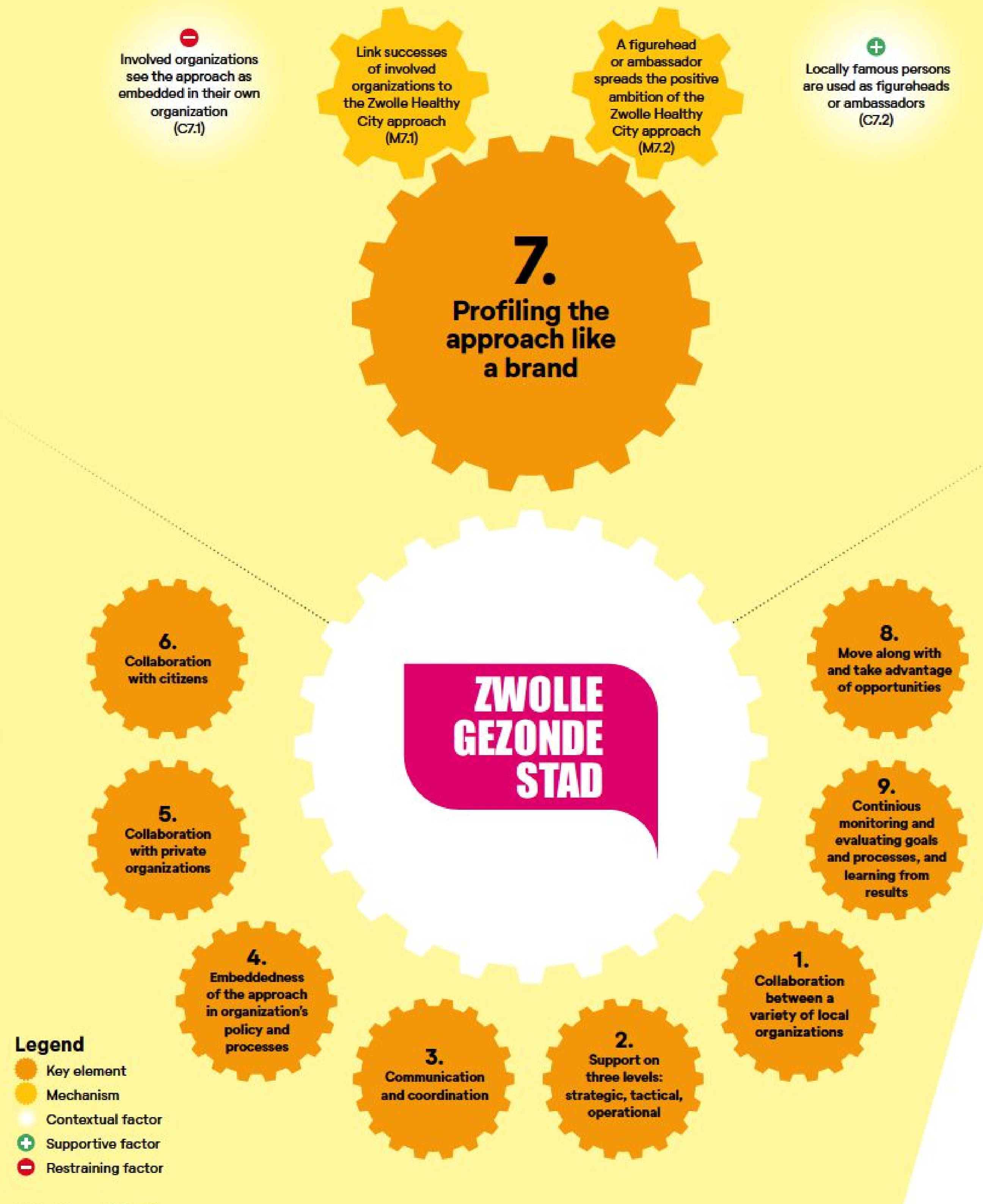

Wilderink et al. (2022)

8. Move along with and take advantage of opportunities

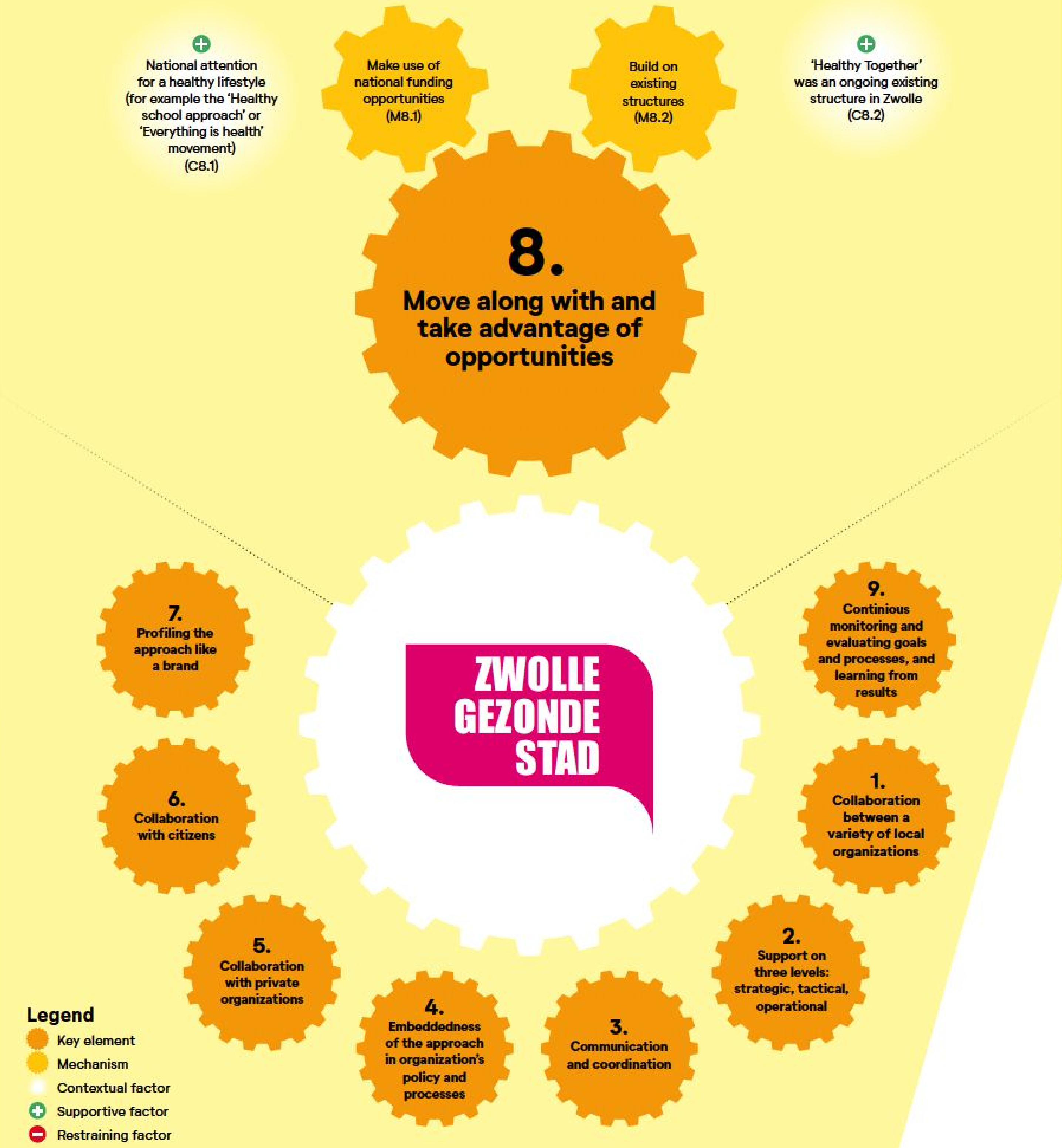

Wilderink et al. (2022)

9. Continious monitoring and evaluating goals and processes, and learning from results

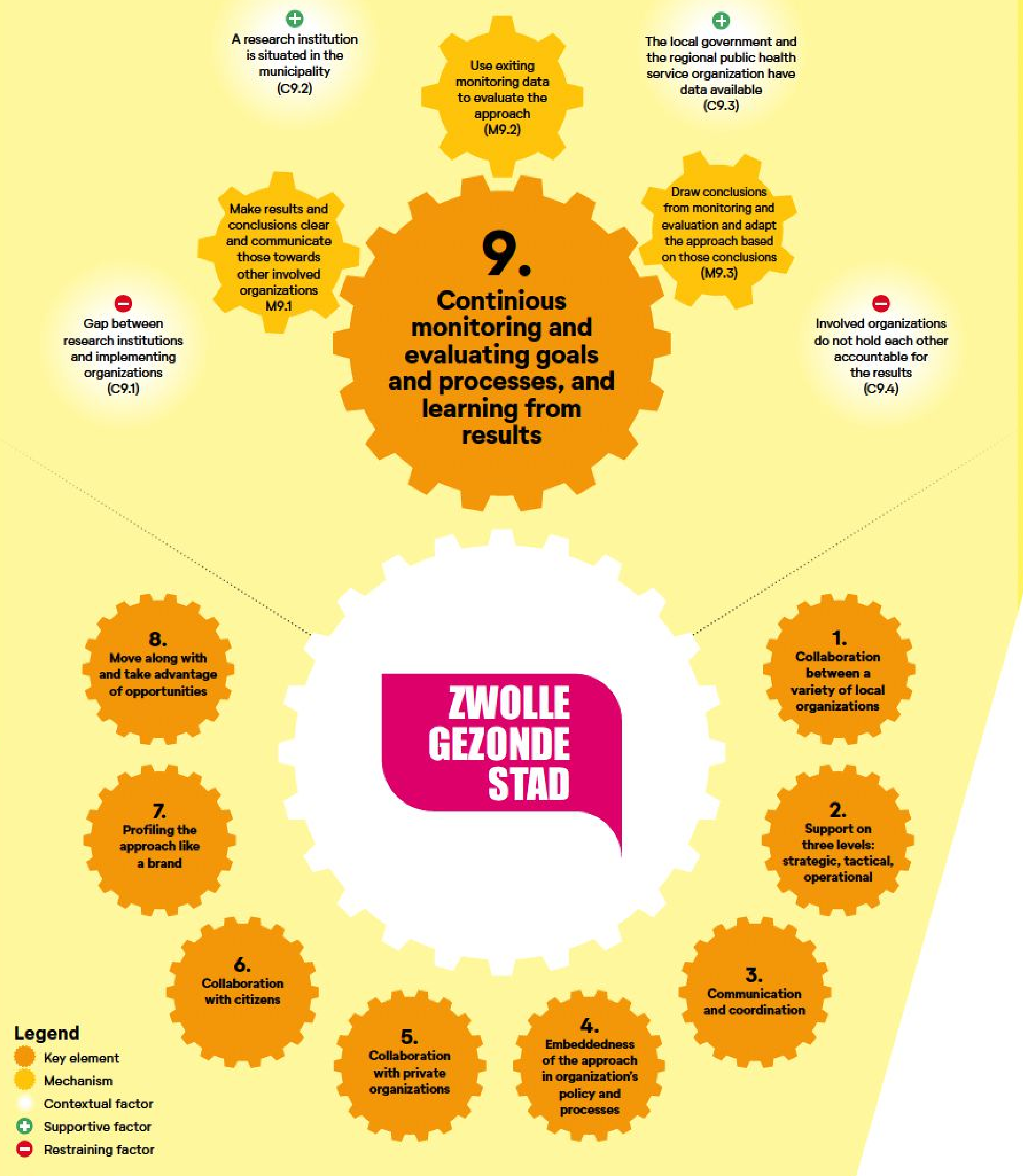

Wilderink et al. (2022)
