# Supplementary material for: Mechanisms and contextual factors related to key elements of a successful integrated community-based approach aimed at reducing socioeconomic health inequalities in the Netherlands: A realist evaluation perspective
Source: PLoS One. 2023 May 17;18(5):e0284903. doi: 10.1371/journal.pone.0284903 (PMC10191363; doi:10.1371/journal.pone.0284903)
Supplement: Attachment — Submitted filename: Response to reviewers.docx [file pone.0284903.s004.docx]

**Response to reviewers**

| **Reviewer #1** |  |
| --- | --- |
| 1. Dear Authors,   Thank you for the submission. While your paper is very interesting and well written, it is methodologically flawed and for this reason needs complete revision and reanalysis.  Firstly, it is not a realist evaluation. Secondly, while you attempted to apply realist data analysis techniques, this does not appear to be done in line with realist principles of generative causation and retroduction.  Best of luck with the rest of the journey! | Dear reviewer #1,  Thank you for taking the time to read and review our manuscript. Considering your and reviewer 2’s feedback, we agree that our study is indeed not a strict realist evaluation study, and that using the term ‘realist synthesis’ was not correct. We used however a realist evaluation *perspective* in the analysis of our qualitative data. Our aim was to answer questions as ‘how does the intervention work’ and ‘in what context does it work’, instead of the question ‘does it work’ which is usually answered in a normal evaluation. These are elements of a realist evaluation. Using such a realist perspective helped us in answering these questions by identifying mechanisms and contextual factors in the primary qualitive data.  We made this more clear in the text by deleting ‘realist evaluation’ from the title of the paper and replacing phrases in the text as ‘using realist evaluation’ for ‘using elements of a realist evaluation perspective’. We also made more clear in the text that we used primary qualitative data.  Moreover, we agree that the realist principle of retroduction and generative causation could be worked out better in the description of mechanisms in the repository and we have made changes accordingly. Mechanisms are not actions or activities themselves, but the underlying processes that made that these actions or activities lead in a certain context to a certain outcome. It’s about the response of the persons involved. Following realist evaluation principles, these actions or activities produce one or more psychological, attitudinal, and behavioral changes.  Therefore, the answers to the questions ‘how’ and ‘why’ these processes happen and the responses of involved people to these mechanisms are now made more clear in the description of mechanisms in the repository and in the main text. |
| **Reviewer #2** |  |
| 1. Overall this is an interesting paper, but there are a number of methodological issues in the manuscript: | Dear reviewer #2,  Firstly, thank you for taking the time to read our manuscript and providing feedback. |
| 2. You state (line 101 and elsewhere) that you used realist synthesis to analyse qualitative data. This may be confusing to people less familiar with realist thinking, who see realist synthesis as very distinct from evaluation and focused on the synthesis of literature rather than the analysis of primary data. I would simply say that you have applied a realist logic of analysis to the qualitative data collected. | Considering your and reviewer 1’s feedback, we indeed believe that using the term realist synthesis is not suitable.  We replaced phrases in the text as ‘using realist evaluation’ for ‘using a realist evaluation perspective’ and ‘realist synthesis’ for ‘qualitative data-analysis with a realist perspective’, because we believe this is more appropriate. We made clearer in the text that we used primary qualitative data. Also, we deleted ‘realist evaluation’ from the title of the paper.  Realist evaluation as an approach is method-neutral as both quantitative as qualitative data can be collected. Identifying observed contextual factors and mechanisms can be done by coding qualitive data. |
| 3. Your definition of mechanisms is erroneous. Mechanisms are not the 'things' introduced by an intervention, but the responses that respondents might have to those, in context. They are the invisible forces that drive change, such as fear, sense of belonging etc. Some of the literature describe mechanisms as the combination of resources introduced by an intervention, and the reasoning of actors as a result of this introduction. Others place the resource within the context. Either way, the element that distinguishes mechanisms from other constructs is that they are intangible and linked to the reactions and thinking of actors and systems. You do however talk about these mechanisms, in their true sense, in the text, but the cross references (Mx.x) are beside the tangible aspects of the intervention. | We changed the operational description of what a mechanism is in table 1, and rephrased the mechanisms in the repository in a way that according to us is still in line with the analyzed data and the correct definition that you describe. We believe that the reactions and thinking of actors was already present in the elaboration on the mechanisms in the repository, but not in the sentence that describes the mechanisms itself. We now changed these sentences in a way that is more in line with your feedback and realist principles. We also did this in the main text in the result section.  In additions to the ‘things’ introduced by the intervention we added to the mechanisms in the repository how this led to reactions and response of involved professionals and how this in combination with the context led to the described outcome. |
| 4. You present the findings per outcome category. Perhaps this could have been mapped out a bit better, if you had given an overview of all outcomes at each level of the system to situate the reader better. However more problematically, some of the text under outcomes contain only mechanisms. Without understanding of the context that facilitated the triggering of that M, it is difficult to distil the originality of the data. Disaggregating C from M does not make a lot of sense in realism. You make that point very well in the section on consulting experts - any one context can be both facilitative or constraining; it depends on the mechanism and therefore it is counter intuitive to separate them. | We indeed chose to present the findings per outcome category. We did not choose to provide an overview of the outcomes for every level, because some outcomes are relevant for multiple levels. However, we made more clear by underlining in the description for what level every specific mechanisms is relevant. We hope this helps in guiding the reader better.  In the original manuscript three mechanisms were described without context (M2.7, M4.2 and M5.2). We understand that only the combination of C, M and O is relevant in realist evaluation. In these three specific cases, no contextual factors were explicitly mentioned by the respondents. We as authors however do believe that these mechanisms should be displayed, because they occurred in the policy context in which the program Zwolle Health City was implemented. Therefore, to meet your feedback we added ‘local policy aims to reduce socioeconomic health inequalities’ as contextual factors to these three mechanisms, because these mechanisms were triggered by the context of implementation of the Zwolle Healthy City approach. |
| 5. In realist evaluation, the point of the CMO configuration is that a specific M interacts with a specific C to lead to a specific O. It is difficult to see how this is applied when you have 6 Ms and 7 Cs to one O. I think your overall logic of analysis is realist, but it is not expressed in a way that is consistent with realist thinking. | We agree that our study is indeed not a strict realist evaluation study. As you mention, we used indeed the realist logic in the analysis of qualitative data. Our aim was to answer questions as ‘how does the intervention work’ and ‘in what context does it work’, instead of the question ‘does it work’ which is commonly answered in regular evaluation studies. This is in line with realist evaluation. Using a realist perspective helped us in answering these questions by identifying mechanisms and contextual factors in the primary qualitive data.  For this reason, we replaced phrases in the text as ‘using realist evaluation’ for ‘using a realist evaluation perspective’ and ‘realist synthesis’ for ‘qualitative data-analysis with a realist perspective’, because we believe this is more suitable. Also, we deleted ‘realist evaluation’ from the title of the paper. |
| 6. I do like the fact that you are presenting your findings at three levels of the system; this is entirely coherent with realist thinking. The discussion is strong and well written. | Thank you. |
