# Supplementary material for: Mechanisms and contextual factors related to key elements of a successful integrated community-based approach aimed at reducing socioeconomic health inequalities in the Netherlands: A realist evaluation perspective
Source: PLoS One. 2023 May 17;18(5):e0284903. doi: 10.1371/journal.pone.0284903 (PMC10191363; doi:10.1371/journal.pone.0284903)
Supplement: Attachment — Submitted filename: Response to reviewer.docx [file pone.0284903.s005.docx]

**Rebuttal 2**

Reviewer #1:

*Dear Authors,

Again thank you for the opportunity to review your paper, again on a very important topic. However, there are still numerous methodological flaws (or, an understanding of the realist perspective) that are present. Reviewing your changes, there were very minimal changes to any content in regards to the methodology, only wording which rephrased realist evaluation to realist evaluation logic or perspective. However, it is unclear how you followed realist evaluation logic or a perspective. Realist evaluation logic at minimum would, I believe, include some component of theory generation and refinement.

You possibly applied realist lens to your analysis. However, this too was not always clear and appears to miss core components of generative causation and retroduction, which are two necessary components of any realist analysis.

I cannot, therefore, provide a more detailed review of the paper until these core methodological components are expanded and addressed. Please could you, therefore:*

| **Suggestion reviewer** | **Response authors** |
| --- | --- |
| *Clearly define how this study incorporated realist evaluation logic* | In the introduction as well in the methods section we now describe how the logic model is seen as the initial programme theory of the Zwolle Healthy City approach and this realist evaluation study contributes to theory refinement by using the stakeholder perspective. Stakeholders’ ideas on what mechanisms and contextual factors led to certain outcomes is a refinement and deepening of this initial programme theory.  Also in the discussion section we now describe that this study showed a further (practical) translation of the initial programme theory. We describe in what way this deepening based on stakeholders’ perspectives led to additions and adjustments of the initial programme theory. |
| *Please clearly describe your analysis process, and how exactly this took a realist perspective. Identifying Contexts and Mechanisms alone is not sufficient to be a realist ‘study’. Generative causation is fundamental, and this was not very clear in your paper.* | In the methods section we now added how we took a realist perspective in the data analysis by seeking certain phrases that tell us more about the generative causation relations between C, M and O’s. |
| *Notably, more information on the relationship between the Cs Ms and Os needs to be given, and what these specific elements contribute.* | We agree that the relationship between the Cs Ms and Os was not made clear enough in the previous version. This was because we decided to disentangle the M’s and C’s for every O, and describe them separately. In the results section of this revised version, we describe how the combination of the Cs and Ms led to the Os. |
| *Please review RAMESES II guidelines to get further input to inform your write-up.* | Thank you for the suggestion. An overview of whether and how the different items of the RAMESES II guideline are included in our paper is now added as a supplementary file. |

*I very much appreciate the focus on understanding ‘how does it work’, not just if it works. There are however other approaches to answer this than a realist approach, which again is a clear methodology which requires strong considerations of theory development/refinement, generative causation and retroduction.*
