# Supplementary material for: Mechanisms and contextual factors related to key elements of a successful integrated community-based approach aimed at reducing socioeconomic health inequalities in the Netherlands: A realist evaluation perspective
Source: PLoS One. 2023 May 17;18(5):e0284903. doi: 10.1371/journal.pone.0284903 (PMC10191363; doi:10.1371/journal.pone.0284903)
Supplement: Attachment — Submitted filename: review for ZHC.docx [file pone.0284903.s006.docx]

Thank you for the opportunity to review your interesting manuscript on the important topic of regarding an approach to addressing socioeconomic health inequalities. Please note this is my first review of your paper, but I have read the previous reviewer’s comments and your responses.

It is clear you have made some major revisions to address the Realist positioning of this paper, and thank you for including the RAMSES guidelines checklist. However, there are a few methodological matters than remain unclear in your paper:

| Abstract | You may wish to consider what readers will be most interested in when reading the results section of your abstract – I suspect a summary of the two/three most key CMOCs will be more of a hook that a quantitative number of CMOCs returnend. |
| --- | --- |
| Introduction/background | Thank you for adding a paragraph clarifying that the study intends to refine an existing logic model.  Purpose of the study: why was a Realist approach appropriate? As an earlier reviewer commented – other approaches can be use to address how & why something works – why Realism? It may be useful here to think about why you were interested in context and causation…. Perhaps for those intending to understand how to implement the model elsewhere? |
| Methods | Good practice is to explain why you used the type of data you used & why you invited the participants that you did – how was this best placed to inform the refinement of your programme theory?  In the discussion/limitations section you mention that the interviews were undertaken prior to the design of this study and that this was a limiting factor in understanding contextual factors in the CMOC. Although the explanation of the limitation is well placed in the discussion, the source of the interviews is perhaps a point for the methods section, particularly in light of the usual expectation to describe why the data were best placed to address your realist programme theory refinement. If these data were collected for a different purpose but have been utilised for this secondary purpose in this realist theory building – please state.  Usually the exact number and role of interviewees is the first thing reported in the results section (unless these were secondary data? In which case could be considered part of the ‘setting’).  The analysis section begins by discussing the use of a Braun & Clarke style thematic analysis – I would question whether this is methodologically congruent with the Realist evaluation approach. As you later mention that the interviews were collected originally for a different purpose I was unsure whether this element took place as part of a different research phase/question.  Do check you are using expected Realist terminology and that you are using it correctly throughout. E.g: Retroductive as an inductive/deductive cycle. “demi-regularities” rather than “patterns”.  The use of ‘experts’ to agree CMOCs is a fantastic aid to your rigour. However, by placing the changes/additions made by these experts in your results section rather than your methods section I was left confused whether you considered the final CMOCs to be those made before the experts added their thoughts or after. Assuming that the final CMOCs are those made after the input of the experts I would move the entire section to your methods. Additionally, there is (I think) a typo in the section that needs moving: the sentence states that experts missed two CMOCs, but I think the intention is that the CMOCs identified two CMOCs that were previously missed? |
| Results | There are some detailed CMOCs reported. This demonstrates some thorough analysis and retroductive thinking.  However, you state your intention was refine your initial programme theory. Therefore, you need to discuss whether these findings confirm/disprove/add detail to/other your initial programme theory. Depending on which, this may be a statement near the beginning of your results, or may be something you address under each outcome. One thing I found a little confusing, for example, was whether the outcomes had changed as part of your theory refinement, or whether these had stayed the same and the context/mechanism configuration causing those outcomes had changed. |
| Discussion | The results are placed in the context of wider literature.  Your reflections on initial programme theory might be better placed at the beginning of this section. |
| Throughout | A few times you mention “specifying” the programme theory. This a little confusing. Are you defining an initial programme theory, or are you refining an existing one? If the latter, consider removing/changing this word |
